# Effects of an Eight-Week Concurrent Training Program with Different Effort Character over Physical Fitness, Health-Related Quality of Life, and Lipid Profile among Hospital Workers: Preliminary Results

**DOI:** 10.3390/ijerph18179328

**Published:** 2021-09-03

**Authors:** Txomin Pérez-Bilbao, David García-González, Álvaro Martos-Bermúdez, Sandra Nieto, Teresa del Campo, Margarita Pérez-Ruiz, Alejandro F. San Juan

**Affiliations:** 1 Department of Health and Human Performance, Faculty of Physical Activity and Sport Sciences-INEF, Universidad Politécnica de Madrid, 28040 Madrid, Spain; txomin.pbilbao@alumnos.upm.es (T.P.-B.); davidgarciaglez@yahoo.es (D.G.-G.); alvaromartosbermudez96@gmail.com (Á.M.-B.); 2 Department of Education, Investigation Methods and Evaluation, Faculty of Human and Social Sciences, Comillas Pontifical University, 28049 Madrid, Spain; 3 Department of Occupational Health and Prevention, University Hospital Fundación Jiménez Díaz, 28040 Madrid, Spain; sandra.nieto@quironsalud.es (S.N.); tcampo@fjd.es (T.d.C.); 4 Research Group on Exercise, Health and Applied Biomarkers, Faculty of Sport Sciences, European University of Madrid, 28670 Madrid, Spain

**Keywords:** concurrent training, exercise, strength, effort character, hospital workers, health

## Abstract

Background: The “effort character” (EC) is a resistance training method without reaching muscle failure. It was defined by González-Badillo and Gorostiaga Ayestarán (2002) as the relationship between the repetitions performed and the repetitions achievable. Then, the EC is at its maximum (i.e., 100%) when the subject realizes all the repetitions possible in a series with any load. Therefore, an EC of 50% indicates execution of 50% of the repetitions achievable in a series. This study aimed to determine the effects of two programs of eight weeks of concurrent training (CT) with different EC over muscle strength (MS), cardiorespiratory fitness (CRF), functional mobility (FM), health-related quality of life (HRQoL), and lipid profile (LP) among hospital workers. Methods: Fourteen hospital workers (age: 41.1 ± 10.8 years; body mass: 63.0 ± 10.8 kg; height: 165.2 ± 6.5 cm; body mass index (BMI): 23.0 ± 3.4 kg/m^2^) were randomly assigned to an EC 50% (*n* = 7) or EC 100% (*n* = 7) group. Results: The main finding was that both groups significantly improved in MS and FM levels but not HRQoL, with no statistical differences between EC 50% and EC 100% in adherence and any test despite performing half the volume of the strength workout. Conclusions: An eight-week CT program with different EC (i.e., EC 50% vs. EC 100%) seems to improve the MS and FM levels in hospital workers similarly. These findings could be very useful in health-training practices because of the possibility of planning training loads with half the volume of strength workouts without the loss of any training adaptation.

## 1. Introduction

Nowadays, many companies sponsor exercise programs as a strategy to improve economics and employees’ health outcomes. These exercise programs have shown benefits in health, such as increases in well-being and reductions of health risk factors, and work productivity [1]. These health benefits could be especially important in health care professionals who take care of the rest of the population. However, physical activity levels in healthcare workers are generally low [2,3]. The reasons given by most healthcare workers (i.e., >70%) for not exercising were “no time”, “too tired”, “not accessible”, and “no partner” [3]. Moreover, a study conducted in Taiwan [2] showed approximately 90% of nurses exercised once a week, and none exercised more than 3 times per week. To try to resolve this situation, some previous studies implemented interventions and indicated that exercise improved the fitness levels and health of healthcare workers [3,4,5]. An exercise program implemented in nurses significantly improved strength levels and cardiopulmonary function [3]. These results are similar to the results found by Christensen et al. [4] that focused on 98 female healthcare workers who participated in an intervention program that consisted of diet, physical exercise, and cognitive behavioral training during working hours for one hour weekly. To the best of the authors’ knowledge, research investigating the effects of exercise in healthcare workers is scarce.

Resistance training (RT) is one of the main exercise methods and produces different muscle adaptations depending on the variables that configure the RT stimulus (i.e., load, sets and repetitions, rest duration, exercise type and order, and movement velocity) [6]. The accumulation of repetitions during RT produces progressive muscle fatigue, and if the subject continues with exercise execution, task failure occurs because volitional fatigue arrives with maximum subject stress and with a maximum rate of perceived exertion (RPE) [7]. In this sense, there are some RT studies that suggest that performing repetitions to failure would be necessary to enhance maximal muscle mass and strength gains [8,9,10]. However, other RT studies have shown similar and even higher improvements in strength and athletic performance without reaching muscle failure [11,12,13,14,15] and with diminished muscle damage, fatigue, and time recovery [12,16]. This RT method without reaching muscle failure was defined by González-Badillo and Gorostiaga Ayestarán [17] as “effort character” (EC). EC was defined as the relationship between the repetitions performed and the repetitions achievable. Then, according to the number of repetitions performed below the maximum in a series, the EC will be different. The EC will be maximized (i.e., 100%) when the subject realizes all the repetitions possible in a series with any load. Therefore, an EC of 50% indicates execution of 50% of the repetitions achievable in a series. Further, RT is oriented to improve adherence to exercise in a sedentary population or with low levels of physical activity or fitness in which the physiological and psychological stress produced during RT must be controlled (e.g., high muscle tension, fatigue, delayed onset muscle soreness, and fear of injury). In this sense, RT programs with moderate EC (e.g., EC 50–70%) may enhance the adherence to exercise but without losing the training adaptations of a higher EC program (e.g., EC 90–100%). Then, RT programs with moderate EC must achieve enough training load to improve one’s physical condition, health, and quality of life. The aim of this study was to determine the effects of two programs of eight-week duration of concurrent training (CT) with different EC over muscle strength (MS), cardiorespiratory fitness (CRF), functional mobility (FM), health-related quality of life (HRQoL), and lipid profile (LP) among hospital workers. We hypothesized that both groups will improve similarly and without significant differences in MS, CRF, FM, HRQoL, and LP.

## 2. Materials and Methods

### 2.1. Experimental Approach

Initially, 25 hospital workers from Hospital Universitario Fundación Jiménez Díaz (HUFJD) in Madrid (Spain) were recruited, thanks to a health promotion at workplace program “+Saludables FJD”, as a convenience sample and thus participated in this study (mean ± standard deviation (SD); age: 35.2 ± 11.0 years; body mass: 63.5 ± 10.0 kg; height: 165.8 ± 7.6 cm; and body mass index (BMI): 23.1 ± 3.3 kg/m^2^). Inclusion criteria were: (a) to be a HUFJD worker; (b) to be aged 25–60 years; and (c) to agree to participate with informed consent. Subjects were excluded if they suffered from any clinical condition that limited their participation or could be aggravated by the exercise program. All participants realized a one-session test to measure anthropometric data, CRF, MS, and FM related to activities of daily living. CRF was delineated with a six-minute walk test (6M WT) to predict peak oxygen uptake (VO_2peak_) and MS with the one repetition maximum (RM) value (kg) that was estimated in the lower limbs (leg press machine) and upper limbs (vertical chest press and lateral pulldown machine), whereas FM was determined with the five-repetition sit-to-stand test (FRSTST) and 30-m walk test (30 WT).

The exercise intervention consisted of 60–90 min CT sessions two times per week on non-consecutive days across eight weeks, with the performance of endurance and strength training in the same session. Randomization of the groups was carried out with the random function of Microsoft Office Excel (Microsoft Corporation, Redmond, WA, USA); participants were assigned to EC 100% group (*n* = 12) or EC 50% group (*n* = 13). The endurance training program was performed using a treadmill. Each participant’s endurance training intensity was calculated as the target heart rate (THR) based on the subject’s age and predicted maximum heart rate and resting heart rate according to heart rate reserve (HRR) method [18]: THR = %EI × (HRmax − HRrest) + HRrest; where %EI = percentage of endurance exercise intensity, HRmax = maximum heart rate in bpm, and HRrest = resting heart rate in bpm. HRmax was predicted using the following formula: HRmax = 211 − 0.64 × age (years) [19]. The EI was identical for both groups. During the first two weeks, participants exercised at 60% EI for 10 min the first week and 20 min the second week. They progressed to 65–70% EI the next two weeks, for 20 min during week 3, and 25 min during week 4. In weeks 5 and 6, participants exercised at 70% EI for 25 and 30 min, respectively. During the last two weeks, 7 and 8, exercise intensity was set at 75% EI for 30 min (Table 1).

Participants in the EC 100% group performing CT with the RT workout performed repetitions until concentric failure in all sets of the strength exercises. The EC 50% group performing CT with the RT workout performed 50% of the possible repetitions of the strength exercises maximally (e.g., five repetitions of 10 RM). During week 1, strength intensity was set at 50% of 1 RM and progressed to 60% of 1 RM in week 2. In weeks 3 and 4, participants exercised at 70% of 1 RM. In weeks 5 and 6, intensity progressed to 75% of 1 RM and increased to 80% of 1 RM in weeks 7 and 8. The number of sets of the strength exercises performed during the intervention were two sets throughout weeks 1–4, progressing to three sets in weeks 5–8. The strength exercises used during the intervention were leg press machine, vertical chest press, lateral pulldown machine, and core exercises in all the sessions. During week 3, a squat machine exercise was added to the strength program. During week 4, a military shoulder press machine exercise was incorporated. Finally, in week 5, a seated cable row machine exercise completed the strength training workout (Table 2).

### 2.2. Participants

From the initial 25 participants of the study, 11 subjects were unable to complete the intervention due to incompatibility with work duties at the hospital (*n* = 10) and muscle injury outside the exercise program (*n* = 1). Then, fourteen subjects were included finally in this study (mean ± standard deviation (SD); age: 41.1 ± 10.8 years; body mass: 63.0 ± 10.8 kg; height: 165.2 ± 6.5 cm; and body mass index (BMI): 23.0 ± 3.4 kg/m^2^). Baseline characteristics of the participants did not significantly differ between the two groups with exception of height, which was higher in the EC 100% group (Table 3).

The study protocol adhered to the ethics guidelines of the Declaration of Helsinki and was approved by the Ethics Committee of the Universidad Politécnica de Madrid (Spain) on 16 September 2019.

### 2.3. Muscle Strength Tests

Before and after eight weeks of the intervention, three exercises were used to estimate 1 RM (kg) of upper (vertical chest press and lateral pulldown machine) and lower (leg press machine) body musculature. All exercises were performed on gym machines.

Three to five days before 1 RM estimation testing, each participant underwent a familiarization session, executing each exercise according to the recommendation of the National Strength and Conditioning Association [20]. During the familiarization session, correct lifting and breathing techniques were taught and practiced using submaximal loads. The practice continued until the subject demonstrated to the researcher proper performance of the movement in every exercise for a total 10 repetitions, using submaximal loads.

During the testing sessions, in order to facilitate recovery and reduce fatigue, the exercises were alternated between upper and lower body. Each subject performed a light warm up for 10 min by running at a self-selected speed on the treadmill, followed by whole-body dynamic stretching exercises. Thereafter, using the load used in the familiarization session, subjects were asked to complete 10 repetitions of each exercise. Afterwards, resistance was progressively increased until the subjects could perform only nine or fewer repetitions of each exercise until muscle fatigue occurred. Three minutes of rest were allowed between each attempt and five minutes between each exercise. No more than five attempts were necessary in any occasion. Brzycki’s 1 RM prediction equation was used to estimate 1 RM based on the resistance and repetitions recorded. The equation is mathematically expressed as PREDICTED 1 RM = W/[1.0278 − 0.0278(R)], where W is the weight use, and R is the number of repetitions performed [21].

### 2.4. Cardiorespiratory Test

CRF was delineated with the 6M WT, a validated test used in the healthy population [22,23,24]. The 6M WT is a self-paced test in which participants are asked to walk laps of a straight, 30-m corridor as quickly as possible for six minutes. Every minute, researchers encouraged participants to continue walking and informed them of the time elapsed. After the test, the participant’s walking distance was measured and recorded. A Polar^®^ S625XTM heart rate (HR) monitor (Polar Electro OY, Kempele, Finland) was used to record HR at the end of the test. Peak VO_2_ was calculated with the following equation for men and women: VO_2peak_ for men = 110.546 + (0.063 × distance covered) − (0.250 × age) − (0.486 × BMI) − (0.420 × height) − (0.109 × HR); and VO_2peak_ for women = 22.506 − (0.271 × body mass) + (0.051 × distance covered) − (0.065 × age) [22].

### 2.5. Functional Mobility Tests

Functional mobility was determined with two tests related to activities of daily living: (1) the FRSTST and (2) the 30-m walk test (30 WT). The FRSTST required participants to stand up from and sit down on a slightly padded, 43-cm-high armless chair as quickly as possible five times. Participants folded their arms across their chests and were instructed to stand up completely and make firm contact with their back against the backrest of the chair when sitting. Timing began on the command “go” and ceased when the participant’s buttocks reached the seat following the fifth stand. Participants were allowed a practice trial of two repetitions before the timing of two test trials of five repetitions. Between trials, a recovery of 60 s was provided [25].

During the 30 WT, participants walked 30 m in a flat corridor as fast as possible. The test consisted of walking the 30 m at maximum walking speed. The test started on the command “go” and ended when the participant crossed the finish line. Two trials were conducted with a two-minute rest period between trials [26]. The fastest of the two test trials was used in subsequent analyses of both tests. Performance time in all the tests was measured by the same investigator with the same stopwatch to the nearest 0.1 s.

### 2.6. Quality of Life

Health-related quality of life (HRQoL) was assessed with the Spanish Short Form 12, version 2 (SF-12v2). The SF-12 is a HRQoL questionnaire consisting of twelve questions that measure eight health domains to assess physical and mental health. The eight health domains include: (1) physical functioning (PF): to perform physical activities without limitations; (2) role physical (RP): problems with daily activities due to physical health problems; (3) bodily pain (BP): presence of pain and its interference in daily activities; (4) general health (GH): subjective evaluation of overall health; (5) vitality (VT): presence of fatigue or loss of energy; (6) social functioning (SF): limitations in social activities; (7) role emotional (RE): difficulties with daily activities due to emotional problems; and (8) mental health (MH): presence of depressive/nervous feelings. These eight domains can be weighted and summarized in two component scores, a physical component score (PCS) and mental component score (MCS). The items from PF, RP, BP, and GH are primarily indicators for PCS, while VT, SF, RE, and MH are primarily indicator for MCS. The PCS and MCS were obtained used the standard scoring algorithm provided by the instrument´s developer [27].

### 2.7. Lipid Profile

Drawn blood samples were collected in Vacutainer^®^ serum-separator tubes, allowing the blood to clot 30–60 min at room temperature and separating by centrifugation for 15 min at 2200–2500 RPM. Serum was clear and free from all red cells. The samples were maintained at 2–8 °C while handling. Laboratory tests were performed by colorimetric enzymatic methods for the quantitative determination of total cholesterol, high-density lipoprotein (HDL) cholesterol, and triglycerides in serum (Roche/Hitachi Cobas c systems). Low-density lipoprotein cholesterol (LDL-C) was calculated using the Friedewald equation: total cholesterol (TC)—(non-high-density lipoprotein cholesterol [non-HDL-C])—(triglycerides [TG]/5) [28].

### 2.8. Statistical Analysis

All statistical analyses were performed with the IBM SPSS-v25 package for Windows (IBM Corporation, Armonk, NY, USA). The Shapiro–Wilk test was used to assess the normality of the data. The primary analysis was a one-way ANCOVA between the exercise intervention group with post-intervention values as the dependent variables and baseline values of each parameter as the covariate. Post-hoc tests with Bonferroni correction were performed as required. A paired *t*-test was used to assess the change between baseline and post-intervention values within each intervention group. Differences between intervention groups at baseline were assessed using independent *t*-test.

Effect sizes (ES) were calculated using Eta squared (η^2^), and their interpretation was based on the following criteria: 0.01 ≤ ES < 0.06 small effects, 0.06 ≤ ES < 0.14 moderate effects, and ES ≥ 0.14 large effects [29]. The intra-group effect size (ES) was calculated using Cohen’s d statistic and was classified as follows: above 0.8: large; between 0.8 and 0.5: moderate; between 0.5 and 0.2: small; and lower than 0.2: trivial [29]. Means (M) and standard deviations (SD) of the variables were calculated for descriptive statistics. The level of significance was set at 0.05.

## 3. Results

Results for MS, CRF, FM, HRQoL, and LP of both groups are shown in Table 4, Table 5 and Table 6. No significant differences for any variable were detected between the training groups at baseline.

### 3.1. Adherence to Training Interventions

Attendance of exercise intervention for participants in the EC 50% and EC 100% groups were 81.3% and 63.4%, respectively. There was no statistically significant difference in adherence levels between the groups (*p* = 0.265).

### 3.2. Muscle Strength

MS significantly increased across time in EC 50% for leg press machine (*p* = 0.001; d = 0.74), vertical chest press (*p* = 0.008; d = 0.38), and lateral pulldown machine (*p* = 0.004; d = 0.45) and in EC 100% for leg press machine (*p* = 0.007; d = 0.61), vertical chest press (*p* = 0.024; d = 0.38), and lateral pulldown machine (*p* = 0.014; d = 0.31). No significant differences were found between EC 50% and EC 100% after eight weeks of CT for leg press machine (F_1,11_ = 1.76; *p* = 0.211; η^2^ = 0.14), vertical chest press (F_1,11_ = 0.45; *p* = 0.516; η^2^ = 0.04), or lateral pulldown machine (F_1,11_ = 2.12; *p* = 0.173; η^2^ = 0.16).

### 3.3. Cardiorespiratory Fitness

Regarding peak VO_2_, EC 50% (*p* = 0.015) but not EC 100% (*p* = 0.400) displayed a significant change in VO_2peak_ during the exercise intervention; in addition, the EC 50% group showed a moderate ES (d = 0.64). After the intervention, no significant changes were found between EC 50% and EC 100% for VO_2peak_ (F_1,11_ = 0.93; *p* = 0.355; η^2^ = 0.08).

### 3.4. Functional Mobility

After eight weeks of CT among hospital workers, participants of EC 50% significantly increased performance of the FRSTST (*p* = 0.007; d = 1.20) and 30 WT (*p* = 0.004; d = 0.68) over time. On the other hand, EC 100% participants significantly improved for FRSTST (*p* = 0.012; d = 1.76) but not for 30 WT (*p* = 0.080). At the end of the intervention, no significant differences arose between EC 50% and EC 100% for FRSTST (F_1,11_ = 0.47; *p* = 0.507; η^2^ = 0.04) or 30 WT (F_1,11_ = 0.07; *p* = 0.802; η^2^ = 0.01).

### 3.5. Health-Related Quality of Life

Table 5 shows the scores on HRQoL. None of the groups showed significant changes in PCS or MCS after eight weeks of intervention: EC 50% (*p* = 0.256 and *p* = 0.099) nor EC 100% (*p* = 0.125 and *p* = 0.240). After intervention, no significant differences were found between EC 50% and EC 100% for PCS (F_1,10_ = 0.19; *p* = 0.676; η^2^ = 0.02) or MCS (F_1,10_ = 1.72; *p* = 0.219; η^2^ = 0.15).

### 3.6. Lipid Profile

After eight weeks of intervention, LDL-C levels significantly decreased in EC 50% (*p* = 0.013; d = 0.48), and HDL-C values significantly increased in EC 100% (*p* = 0.021; d = 0.24). No significant differences were found between EC 50% and EC 100% after eight weeks of CT for TC (F_1,11_ = 1.70; *p* = 0.218; η^2^ = 0.13), LDL (F_1,11_ = 0.19; *p* = 0.668; η^2^ = 0.02), HDL (F_1,11_ = 1.05; *p* = 0.327; η^2^ = 0.09), or TG (F_1,11_ = 0.13; *p* = 0.729; η^2^ = 0.01).

## 4. Discussion

The present study examined the adaptations in MS, CRF, FM, HRQoL, and LP for two different eight-week CT programs. The main findings were that both groups significantly improved MS and FM levels but not HRQoL. Moreover, the EC 50% group improved CRF and the LDL-C values of the LP. The EC 100% group improved HDL-C values of the LP. No statistical differences between EC 50% and EC 100% were found in any test despite the fact that EC 50% performed half the volume of the strength workout. However, it is possible that the low adherence of EC 100% (63.4%) may have limited intra- and inter-group differences. To our knowledge, this is the first study investigating CT with different effort character over strength and FM among hospital workers.

In terms of adherence to the exercise program, there were no statistical differences between the EC 50% and EC 100% groups. However, it seems there was a positive trend in the EC 50% group adherence (+17.9%) that may have been due to the better affordable load planification (i.e., half the volume training repetitions). This issue should be deeply investigated because low exercise adherence is one of the biggest problems in hospital workers [15] and a main limitation factor in the promotion of exercise’s positive benefits and adaptations in health and quality of life.

Our global results are consistent with the findings of Yuan et al. [15], who implemented an aerobic training program three to five times a week during three months for nurses. They observed significant improvements on hand grip strength, endurance strength of abdominal and back muscles, hamstring flexibility, cardiopulmonary function, and BMI. In comparison, Brox and Froystein [30] performed a weekly exercise class (i.e., light aerobic exercise, muscle strengthening, and stretching) during a six-month period in employees of a community-based nursing home for the elderly. No differences between the intervention and control groups were found for aerobic fitness, HRQoL, or sickness absence. These results may be due to the low exercise frequency (i.e., once weekly) and the low adherence to the intervention (less than 50%) [30].

Other multidimensional interventions that included exercise programs showed positive effects in healthcare workers. Tveito and Eriksen [17] conducted an integrated health program (i.e., physical exercise, stress management training, health information, and ergonomic evaluation of workplace) twice weekly during working hours for nurse personnel. The intervention group reported improvement in health, physical fitness, muscle pain, stress management, maintenance of health, and work situation. Furthermore, Christensen et al. [16] realized an intervention of 12 months consisting of diet, physical exercise, and cognitive behavioral training during working hours for one to two hours per week in overweight female healthcare workers. The intervention group significantly reduced body mass, BMI, and body fat percentage, but there were no changes in CRF or MS.

To our knowledge, there are no eight-week exercise intervention studies in hospital professionals to which we can compare our results. However, Varela-Sanz, Tuimil, Abreu, and Boullosa [31] performed an eight-week concurrent training intervention in 35 moderately active sport sciences students and obtained an increase in maximal strength of 17.1% and 40.4% in bench press and half squat, respectively, and a 4.3% increase in VO_2peak_ levels. Moreover, Fyfe, Bartlett, Hanson, Stepto, and Bishop [32] applied a concurrent exercise program during eight weeks in 23 recreationally active men and observed improvements of 13.9% and 25.8% in bench press and leg press strength, respectively, and a 4.6% increase in VO_2max_. In these exercise intervention studies [31,32], strength and VO_2peak_ levels improved similarly to the present study, with gains in EC 50% and EC 100% groups in vertical bench press of 19.3% and 28.5%, respectively; leg press 46.8% and 34.2%, respectively; and VO_2max_ improved by 6.3% in EC 50% but only by 1.2% in EC 100%.

RT strength improvements at 1 RM after the eight-week CT program were significant across time with moderate ES values. In the upper limbs with vertical chest press exercise, the EC 50% group showed improvements of +19.3% (ES = 0.38), and the EC 100% group showed +28.5% improvement (ES = 0.38), and with lateral pulldown machine exercise, EC 50% improved +17.5% (ES = 0.45) and EC 100% improved +9.5% (ES = 0.31). Moreover, in lower limbs, the EC 50% showed gains in leg press machine exercise of +46.8% (ES = 0.74), and EC 100% showed improvements of +34.2% (ES = 0.61). Regarding 1 RM values, the improvements observed in the present study are consistent with previous studies investigating CT effects in untrained elderly subjects [33] and office workers [34] following similar training programs. However, our results showed that performing repetitions to concentric failure did not provide additional strength increases even in the EC 50% group, who performed strength training workouts with the same intensity (e.g., 70% 1 RM) but with half the volume (e.g., six repetitions over 12 RM) compared to the EC 100% group. Strength training based on repetitions to concentric failure implied longer training sessions, higher neuromuscular fatigue, higher RPE, and discomfort [8,12,35]. In addition, repetitions to concentric failure should not be performed repeatedly over long periods due to the high potential for overtraining and overuse injuries [36]. Therefore, we believe this type of ST training is not necessary to improve strength gains in this population.

In the present work, most of the MS, CRF, and FM tests reached statistical significance after the intervention in both groups. Most of the tests performed showed moderate to large ES, as displayed in Table 4. In the rest of the variables in which we did not find significant differences (i.e., HRQoL and LP), we must consider that the participants in our study were healthy subjects, and it could be difficult to obtain significant improvements in only eight weeks. This type of intervention might be more beneficial in unhealthy subjects usually associated with low physical activity levels in which smaller increases in physical activity may produce equal or higher health benefits than in healthy and physically active people [37].

Regarding CRF, only the EC 50% group experienced significant improvement after exercise intervention (+6.3%), with a moderate ES (ES = 0.64). Several studies have measured CRF in both healthcare [15,38] and non-healthcare workers [34,39]. All of the interventions showed an improvement in CRF, ranging from 7.2–12.8%. A possible explanation for the low improvement in our study could be that the documented durations of these interventions were considerably longer than ours. These studies were conducted for at least four months and up to a maximum of 12 months, whereas our intervention only lasted eight weeks. This fact, together with our small sample size, could explain the low improvement in CRF.

The exercise program did not significantly increase FM assessed by 30 WT in EC 100% (*p* = 0.080); however, the ES in the EC 100% group was large (ES = 0.90) compared to the EC 50% group (ES = 0.68). The FRSTST significantly improved in both groups, with large ES (EC 50% group, ES = 1.20; EC 100%, ES = 1.76). Different studies have measured FM, employing FRSTST and 30 MT in healthy young adults and in a clinical population as well [25,26,40]. According to the age-band specific category for FRSTST provided by Bohannon et al. [25], our results were 3.3% and 6.2% faster in the EC 50% group and in the EC 100%, respectively. With respect to the 30 WT, reference values for healthy Swedish people aged 40–79 years are 1.16–1.47 m/s [41]. As a result, maximal speeds were higher in our study, although our participants were overall younger.

None of the training programs showed significant differences throughout the eight weeks of intervention in either of the two HRQoL parameters (i.e., PCS and MCS). Nevertheless, the EC 50% group showed a decrease in MCS with moderate ES (0.61). In comparison, the EC 100% group improved in PCS with a moderate ES (0.56). Our results agreed with those found in the review conducted by Nguyen et al. [42], in which most of the randomized control trials conducted in healthy office workers reported no differences in HRQoL scores.

Regarding the LP, both the EC 50% and EC 100% groups showed a positive trend in most of the LP variables. The EC 50% group showed a significant improvement in LDL-C values (ES = 0.48) and near-moderate ES in TC (0.50) values. In addition, the EC 100% group showed a significant enhancement in HDL-C values (ES = 0.24). However, it increased TG levels after eight weeks of CT (ES = 0.69), but was always between healthy levels. There is ample evidence demonstrating the effectiveness of both aerobic exercise and resistance exercise in improving LP in different populations [43]. However, in the case of CT, the evidence is limited, and the results are contradictory, with some research documenting significant improvements in LP and others not [44]. In the study conducted by Shaw et al. [45], LDL-C was significantly reduced after 16 weeks of CT in 28 healthy male subjects. However, LeMura et al. [46] found no significant differences in TC, HDL-C, LDL-C, or TG after 16 weeks of CT. Our participants were healthy subjects with values within the normal ranges at the beginning and end of the intervention. This circumstance and the fact that the intervention lasted only eight weeks may explain why some values improved in a preventive but not statistically significant way.

Finally, lower-level exercise training strategies, such as the EC 50% used in the present study, might not cause acute inflammation by muscle damage but could activate the intracellular quality control system and may prevent chronic inflammation [45]. Moreover, the maintenance and enhancement of muscle strength by exercise can prevent and improve the metabolic syndrome, the age-related diseases, and the cellular immune function and resistance against infections and cancer [47].

Our study has limitations that need to be addressed. First, the small sample size affected statistical power, which limited the ability to detect significant differences in several outcome measures. Second, average to poor adherence (i.e., 81.3% for EC 50% and 63.4% for EC 100%) could have contributed to worse outcomes. Third, cardiorespiratory performance could be assessed with direct methods (i.e., gas analyzer) to ensure more accurate values. Finally, the lack of a control group weakens our capacity to draw conclusions.

## 5. Conclusions

An eight-week concurrent training program seemed to improve muscular strength and functional mobility levels in hospital workers. The different effort characters between groups (i.e., EC 50% vs. EC 100%) did not show statistical differences on these results. To our knowledge, this is the first study investigating concurrent training with different effort character over health and quality of life among hospital workers.

These findings could be very useful in health training practices because of the possibility of planning training loads with half the volume of strength workouts without the loss of any training adaptation. Moreover, this plan strategy may enhance adherence to the exercise program and diminish the injury risk associated with strength training.

## Figures and Tables

**Table 1:** Progression of cardiorespiratory workout throughout the 8-week training intervention.

Week	Duration (min)	Intensity (%EI)	CR-10
1	10	60	5
2	20	60	5
3	20	65–70	5–6
4	25	65–70	5–6
5	25	70	6
6	30	70	6
7–8	30	75	7

CR-10, category-ratio scale of perceived exertion-10; %EI, percentage of endurance exercise intensity.

**Table 2:** Progression of resistance training throughout the 8-week training intervention.

Training Group	Week 1	Week 2	Week 3	Week 4	Week 5	Week 6	Week 7	Week 8
EC 50% Group								
Sets × repetitions	2 × 10	2 × 8	2 × 6	2 × 6	2 × 5	3 × 4–5	3 × 4	3 × 4
Rest (s)	30	30	30	30	30	30	30	30
No. of exercises	3	3	4	5	6	6	6	6
% 1 RM	50	60	70	70	75	75–80	80	80
EC 100% Group								
Sets × repetitions	2 × 15–20	2 × 15	2 × 12	2 × 12	2 × 10	3 × 8–10	3 × 8	3 × 8
Rest (min)	30	30	30	30	30	30	30	30
No. of exercises	3	3	4	5	6	6	6	6
% 1 RM	50	60	70	70	75	75–80	80	80

1 RM, one repetition maximum.

**Table 3:** Characteristics of the participants.

Variables	EC 50% (*n* = 7)	EC 100% (*n* = 7)	*p*-Value
Age (years)	41.44 ± 9.80	40.80 ± 12.59	0.917
Female/male (*n*)	5/2	5/2	N/A
Body mass (kg)	61.66 ± 9.14	64.36 ± 12.79	0.658
Height (m)	1.62 ± 0.03	1.69 ± 0.08	0.046 *
BMI (kg·m^−2^)	23.50 ± 3.08	22.58 ± 3.87	0.630

Abbreviations: EC 50%, effort character of 50%; EC 100%, effort character of 100%; BMI, body mass index. Data are presented as mean ± standard deviation; * significant (*p* < 0.05).

**Table 4:** Results of peak oxygen uptake, muscle strength, and functional mobility.

Variables	Group	PRE	POST	EC 50% vs. EC 100% (*p*) *	Intra-Group ES (d)
FRSTST (s)	EC 50%	8.20 ± 0.44	7.35 ^a^ ± 0.98	0.507	1.20
	EC 100%	8.52 ± 0.61	7.13 ^a^ ± 0.97		1.76
30 WT (m·s^−1^)	EC 50%	2.80 ± 0.26	3.01 ^a^ ± 0.29	0.802	0.68
	EC 100%	2.72 ± 0.20	2.90 ± 0.35		0.90
VO_2peak_ (mL·kg^−1^·min^−1^)	EC 50%	42.46 ± 3.22	45.14 ^a^ ± 5.21	0.355	0.64
	EC 100%	42.13 ± 6.72	42.65 ± 8.14		0.07
1 RM LPM (kg)	EC 50%	78.72 ± 41.93	115.57 ^a^ ± 57.90	0.211	0.74
	EC 100%	78.87 ± 36.21	105.83 ^a^ ± 52.58		0.61
1 RM LPM/BM	EC 50%	1.23 ± 0.52	1.80 ^a^ ± 0.68	0.182	0.95
	EC 100%	1.19 ± 0.41	1.57 ^a^ ± 0.65		0.72
1 RM VBP (kg)	EC 50%	44.58 ± 20.35	53.17 ^a^ ± 24.98	0.516	0.38
	EC 100%	40.08 ± 27.33	51.50 ^a^ ± 32.47		0.38
1 RM VBP/BM	EC 50%	0.70 ± 0.24	0.83 ^a^ ± 0.28	0.666	0.50
	EC 100%	0.60 ± 0.33	0.75 ^a^ ± 0.36		0.44
1 RM LPD (kg)	EC 50%	47.27 ± 18.11	55.53 ^a^ ± 18.42	0.173	0.45
	EC 100%	45.61 ± 13.55	49.96 ± 14.69		0.31
1 RM LPD/BM	EC 50%	0.75 ± 0.19	0.88 ^a^ ± 0.17	0.100	0.72
	EC 100%	0.71 ± 0.12	0.76 ^a^ ± 0.13		0.40

Abbreviations: 1 RM, one repetition maximum; 30 WT, 30-m walk test; BM, body mass; EC 50%, effort character of 50%; EC 100%, effort character of 100%; FRSTST, five-repetition sit-to-stand test; LPM, leg press machine; LPD, lateral pulldown; VO_2peak_, peak oxygen uptake; VCP, vertical chest press. Data are presented as mean ± standard deviation; ^a^ significant within-group change (*p* < 0.05, paired test); * ANCOVA.

**Table 5:** Results of Quality of Life.

Variables	Group	PRE	POST	EC 50% vs. EC 100% (*p*) *	Intra-Group ES (d)
Physical Score	EC 50%	53.63 ± 6.17	55.65 ± 3.83	0.676	0.40
	EC 100%	52.05 ± 6.95	54.65 ± 2.41		0.56
Mental Score	EC 50%	51.40 ± 4.35	46.54 ± 11.50	0.219	0.61
	EC 100%	54.00 ± 3.89	55.33 ± 2.03		0.45

Abbreviations: EC 50%, effort character of 50%; EC 100%, effort character of 100%. Data are presented as mean ± SD; There were no significant differences within groups; * ANCOVA.

**Table 6:** Results of Lipid Profile.

Variables	Group	PRE	POST	EC 50% vs. EC 100% (*p*) *	Intra-Group ES (d)
TC (mg∙dL^−1^)	EC 50%	175.86 ± 14.65	169.43 ± 11.18	0.218	0.50
	EC 100%	189.29 ± 29.80	188.86 ± 29.08		0.02
LDL-C (mg∙dL^−1^)	EC 50%	99.14 ± 17.57	91.14 ^a^ ± 15.93	0.668	0.48
	EC 100%	108.71 ± 22.31	101.57 ± 22.19		0.32
HDL-C (mg∙dL^−1^)	EC 50%	59.43 ± 10.41	61.00 ± 10.60	0.327	0.15
	EC 100%	68.29 ± 19.53	73.29 ^a^ ± 22.33		0.24
TG (mg∙dL^−1^)	EC 50%	86.29 ± 37.53	83.00 ± 26.89	0.729	0.10
	EC 100%	61.43 ± 13.44	71.29 ± 15.30		0.69

Abbreviations: EC 50%, effort character of 50%; EC 100%, effort character of 100%; HDL-C, high-density lipoprotein cholesterol; LDL-C, low-density lipoprotein cholesterol; T, time effect; TC, total cholesterol; TG, triglycerides. Data are presented as mean ± SD; ^a^ significant within-group change (*p* < 0.05, paired test); * ANCOVA.

## Data Availability

The data presented in this study are available on request from the corresponding author.
